# *Bacillus pumilus* B12 Degrades Polylactic Acid and Degradation Is Affected by Changing Nutrient Conditions

**DOI:** 10.3389/fmicb.2019.02548

**Published:** 2019-11-22

**Authors:** Kyle S. Bonifer, Xianfang Wen, Sahar Hasim, Elise K. Phillips, Rachel N. Dunlap, Eric R. Gann, Jennifer M. DeBruyn, Todd B. Reynolds

**Affiliations:** ^1^Department of Microbiology, University of Tennessee, Knoxville, Knoxville, TN, United States; ^2^Department of Biosystems Engineering and Soil Science, Institute of Agriculture, University of Tennessee, Knoxville, Knoxville, TN, United States

**Keywords:** poly-lactic acid, degradation, *Bacillus*, regulation, assay

## Abstract

Poly-lactic acid (PLA) is increasingly used as a biodegradable alternative to traditional petroleum-based plastics. In this study, we identify a novel agricultural soil isolate of *Bacillus pumilus* (B12) that is capable of degrading high molecular weight PLA films. This degradation can be detected on a short timescale, with significant degradation detected within 48-h by the release of L-lactate monomers, allowing for a rapid identification ideal for experimental variation. The validity of using L-lactate as a proxy for degradation of PLA films is corroborated by loss of rigidity and appearance of fractures in PLA films, as measured by atomic force microscopy and scanning electron microscopy (SEM), respectively. Furthermore, we have observed a dose-dependent decrease in PLA degradation in response to an amino acid/nucleotide supplement mix that is driven mainly by the nucleotide base adenine. In addition, amendments of the media with specific carbon sources increase the rate of PLA degradation, while phosphate and potassium additions decrease the rate of PLA degradation by *B. pumilus* B12. These results suggest *B. pumilus* B12 is adapting its enzymatic expression based on environmental conditions and that these conditions can be used to study the regulation of this process. Together, this work lays a foundation for studying the bacterial degradation of biodegradable plastics.

## Introduction

The crisis of plastic pollution in the environment has led to an increased adoption of biodegradable polymers in the commercial plastic industry. Biodegradable polymers are considered a more ecologically friendly alternative to conventional plastic polymers due to a limited accumulation in the environment (Emadian et al., 2017). Some of these biodegradable polymers, such as poly-hydroxyalkanoate (PHA) and polylactic acid (PLA), are derived from renewable biomass (Madkour et al., 2013; Singhvi and Gokhale, 2013), while other biodegradable polymers are synthesized from fossil fuel feed stocks (e.g., PBAT and PCL). Biodegradable polymers like these are polyesters or co-polyesters, formed with ester linkages that are common to many macromolecules that are both synthesized and degraded by microbial life. This is in contrast with conventional petroleum-based polymers that are rich in carbon-carbon linkages which are resistant to microbial degradation, rendering the polymers more recalcitrant.

Polylactic acid is the second most common biodegradable polymer found in commercial plastics (European Bioplastics, 2018). PLA is an aliphatic polyester formed from direct condensation of lactic acid molecules or from ring polymerization of lactide (Castro-Aguirre et al., 2016). Several soil bacteria including *Bacillus brevis* and fungi such as *Rhizopus delemer* have been identified that are capable of degrading PLA, mainly in composting conditions (Fukuzaki et al., 1991; Tomita et al., 1999). This degradation is carried out by a variety of proteases and lipases that hydrolyze PLA to form lactic acid (Oda et al., 2000; Lim et al., 2005; Masaki et al., 2005; Hanphakphoom et al., 2014). Most previous research monitoring biodegradation has focused on the production of CO_2_ as an indicator of cellular respiration and loss of film mass as a gauge for polymer degradation (Ahn et al., 2011; Arrieta et al., 2014; Mihai et al., 2014). This practice tends to be performed over the course of weeks to months and does not lend itself to rapid identification of novel microbial isolates, quick detection of enzymes capable of PLA degradation, or speedy categorization of microbial adaptation through changes in gene expression.

Members of the genus *Bacillus* are spore-forming gram-positive rods which include several species, such as *Bacillus subtilis* and *Bacillus thuringiensis*, that are amongst the most widely studied microorganisms in molecular biology. This genus is well known for the large quantities of secreted and surface associated enzymes they can produce. As a result, *Bacillus* spp. are used heavily in various biotechnological settings (Schallmey et al., 2004; Van Dijl and Hecker, 2013). *Bacillus pumilus* specifically has been shown to be remarkably resistant to many forms of environmental stresses such as intense ultraviolet light and high concentrations of hydrogen peroxide (Link et al., 2004; Handtke et al., 2014). With the combination of high stress tolerance, genetic tractability and high enzyme expression, *B. pumilus* is an ideal candidate for further investigation in polymer degradation.

Although several bacterial and fungal strains have been identified that are capable of degrading PLA, very little is understood about the underlying regulatory mechanisms of degradation. Many of these organisms live in dynamic environments where nutrient conditions are subject to temporal change. In order to develop hypotheses concerning genetic regulation, there is a need to develop degradation assays which align closer with the timescale of microbial adaptation. In addition, fundamental experiments identifying the conditions under which degradation occurs are needed in order to build a mechanistic understanding of cellular-level biodegradation. In this study we identify a novel agricultural isolate of *B. pumilus* (B12) that is capable of degrading high molecular weight PLA films. Our goals were to (1) develop a rapid test for identifying markers of PLA degradation; and (2) determine if changing nutrient supplementation in the media affects PLA degradation rates. Our results suggest that *B. pumilus* B12 is adapting its enzymatic expression based on environmental conditions and that these conditions can be used to study the regulation of this process.

## Materials and Methods

### Microbial Enrichment and Growth Conditions

Soil samples were collected from agricultural soil (sandy loam; 59.9% sand, 23.5% silt, and 16.6% clay; classified as a fine kaolinitic thermic Typic Paleudults) at the University of Tennessee East Tennessee Research and Education Center in Knoxville, TN, United States. Soils were diluted in sterile water, then inoculated onto petri dishes with squares of biodegradable plastic mulch films containing PLA in minimal salt media (MSM) agar, following the methods described in Bailes et al. (2013). MSM was composed of 7.5 mM NH_4_SO_4_, 11 mM KHPO_4_, 2.87 mM K_2_HPO4, 1.7 mM MgSO_4_, and 17.1 mM NaCl, pH 7.0; 1.5% agar was added to make MSM plates. The biodegradable mulch films included BioAgri^®^ (BioBag Americas, Inc.), comprised of Mater-Bi^®^ and an experimental film comprised of a blend of PLA and polyhydroxyalkanotaes (PHA). More details on film composition and properties can be found in Hayes et al. (2017). Soil slurries were plated and incubated statically at 30°C. Single colonies were selected from plates and re-plated on LB agar until morphologically homogeneous colonies were observed, indicative of a pure culture. To test for degradation abilities, strains were inoculated into 100 mL liquid MSM with UV-sterilized 4 cm × 4 cm squares of BioAgri^®^ or PLA/PHA films. Triplicate cultures were grown in sealed flasks with rubber septa for headspace sampling. Flasks were incubated for 16 days at 30°C in a shaking incubator at 180 rpm. Two controls were included in each experiment: one without microbial innocula (i.e., plastic film only) and one without plastic film (i.e., microbes only). Headspace CO_2_ concentrations were determined every 4 days by sampling 0.5 mL headspace gas with a syringe and analyzing on an LI-820 infrared gas analyzer (Licor Inc.) and used as an indicator of plastic film degradation. Plastic pieces were also weighed before and after incubation, and mass loss used as an indicator of degradation. Plastic pieces were rinsed several times with sterile distilled water, dried overnight at 30°C, then weighed to determine mass.

One isolate that exhibited the ability to degrade BioAgri^®^ and PLA/PHA films was identified with 100% gene similarity as *B. pumilus* via 16S rRNA gene sequencing and chosen for further experimentation. *B. pumilus* cultures were grown overnight, washed three times with phosphate buffered saline and diluted to an OD_600__nm_ of 0.1 (approximately 3.125^∗^10^8^ CFU/mL) for all PLA degradation experiments. Cultures were kept static at 30°C in MSM for 48 h. When adding complete supplementation media to liquid cultures, MP Biomedicals^TM^ CSM-HIS-LEU-URA Powdered Media (Fisher-MP114531212) was used. CSM media contains various amino acids and adenine. All carbon supplementation experiments used a concentration of 55 μM for all sugars added to the MSM. When testing nitrate as the main nitrogen source, NaNO_3_ was supplemented in place of NH_4_SO_4_. For growth curve measurements, OD_600_ was measured every 30 min for 72 h in a Biotek Cytation 5 plate reader.

### Phylogenetic Analysis of 16S rRNA Gene Sequences

Genomic DNA from *B. pumilus* was isolated through phenol-chloroform extraction. Universal 16S primers 27F and 1522R were used for PCR sequence amplification. Amplicons were Sanger sequenced at the University of Tennessee Genomics Core. Near full length 16S nucleotide sequences were downloaded from NCBI (Benson et al., 2008). Multiple sequence alignments were performed in MEGA7 (Kumar et al., 2016) using the MUSCLE algorithm (Edgar, 2004). Maximum likelihood phylogenetic reconstruction was performed in MEGA7 with 500 bootstrap replications to determine confidence values for each node. 16S sequence data is found at https://www.ncbi.nlm.nih.gov/nuccore/mn540638.

### PLA Film Creation

Polylactic acid films were created from PLA beads with an inherent viscosity of 1.8 dl/g and a molecular weight between 80,000 and 100,000 kDa (Polysciences, Inc.). For L-lactate detection and scanning electron microscopy (SEM), 10 mg of PLA were dissolved in 1 mL of chloroform, and the dissolved PLA liquid was deposited into autoclaved flat bottom glass vials that are ∼1 cm in diameter. The chloroform was evaporated under nitrogen gas in a fume hood over 1 h resulting in a film that was cast on the bottom of the vial. Vials were stored at −20°C with sterile lids to reduce desiccation.

### L-Lactate Detection Assay

Detection of L-lactate released into the supernatant was performed using ENZYFLUO L-lactate kit (Bioassay Systems Inc.). Overnight cultures were grown in Lennox Broth (LB) shaking at 225 RPM at 30°C and washed three times with phosphate buffered saline. An optical density of 0.1 was incubated in 1 mL of media on 10 mg/mL PLA films in glass vials for 48 h. Supernatant was taken off cultures centrifuged at 13,000 RPM for 2 min from 10 mg/mL PLA films. A 1:1 mixture of supernatant collected from PLA films was added to the L-lactate kit detection solution giving a total volume of 100 μL in a 96-well plate. The supernatant/detection solution was incubated in the dark at room temperature for 1 h and colorimetric changes were measured using a 535/580 nm excitation/emission ratio in a Biotek Cytation 5 plate reader. Results were normalized to a standard curve generated with L-lactate standards ranging from 0 to 42 μM. All measurements were added to the slope generated by the standard curve, yielding approximate concentrations of L-lactate release. All trials included negative controls containing cultures with no PLA added, as well as PLA films in media only. Measurements were taken with 3 biological replicates each with 3 technical replicates, yielding nine total data points per treatment. Statistical significance was determined via *t*-test or one-way ANOVA, as appropriate, with *p*-values below 0.05 considered significant.

### Scanning Electron Microscopy

*Bacillus pumilus* cultures were incubated at 30°C for 48 h with 10 mg/mL PLA films in MSM. Films were then washed three times with phosphate buffered saline (PBS) and fixed with 2.5% glutaraldehyde for 1 h. Samples were then gradually dehydrated with ethanol anhydrous (25, 50, and 100%) and air dried in a fume hood overnight. Samples were mounted on aluminum stubs, sputter coated with iridium and observed using a 1 keV Zeiss GeminiSEM scanning electron microscope.

### Atomic Force Microscopy

Polylactic acid films 50 mg/mL in thickness were incubated for 48 h in MSM. Samples were washed with phosphate buffer saline 5 times to remove excess biomass. Each sample was then imaged using a 5500 PicoPlus atomic force microscope (AFM) operating with the 1.20.2 operating system (Keysight Technologies Inc., Santa Rosa, CA, United States). The instrument was operated in contact mode using MLCT probes with spring constants of 0.01 and 0.03 N/m with a tip radius of 10 nm and Poisson ratio of 0.5. For both imaging and elasticity measurements, the applied force was kept at 3–5 nN. The force distance curves were measured with 8 × 8 points, with each point being an average of 3 force curves. These measurements were recorded on 2-μm^2^ areas on the top of plastic for an average of 5 different locations. This data was then converted to elasticity measurements using the Keysight software in PicoPlus 5500 AFM (Hasim et al., 2017). Statistical significance was determined via *t*-test with *p*-values below 0.01 considered significant. Elastic strength was measured using the Young’s modulus, with values given in kilopascals (kPa).

## Results

### Release of L-Lactate Is Used to Measure Poly-Lactic Acid Degradation by Hydrolases

**FIGURE 1:**
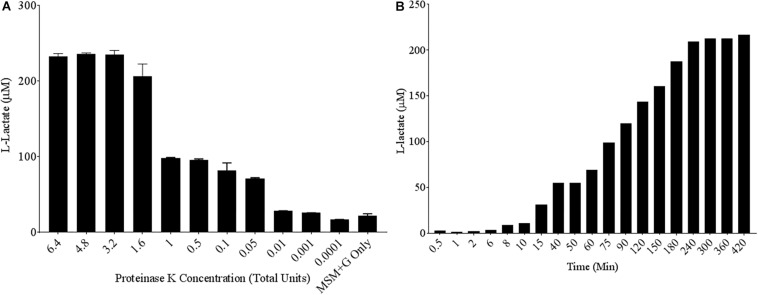
High levels of L-lactate release detected from PLA films exposed to Proteinase K. **(A)** 10 mg/mL Poly-lactic Acid (PLA) films were incubated at 30°C for 24 h with serial dilutions of Proteinase K (*n* = 3). Supernatant samples were collected and added to a colorimetric L-lactate detection kit. MSM + G represents minimal salt media (MSM) with 55 mM glucose supplementation. **(B)** PLA films were incubated with 1.6 total units of Proteinase K with supernatant samples being collected at successive time points.

To quantify poly-lactic acid (PLA) degradation over relatively short time periods, a commercially available L-lactate detection kit was used. A known PLA degrading enzyme, the serine protease Proteinase K, was chosen as a positive control to test the efficacy of the colorimetric assay (Li and Mccarthy, 1999). Poly-lactic acid films of 10 mg/mL were created in 96-well plate formats and serially diluted concentrations of Proteinase K were added to each well in MSM. Following 24 h of incubation at 30°C, up to 100 μM concentrations of L-lactate were detected in the supernatant of PLA samples exposed to Proteinase K (Figure 1A). Proteinase K unit concentrations as low as 0.05 units/mL can release significant concentrations L-lactate compared to the media control over a 24-h period. Since Proteinase K is a useful positive control, we wanted to determine its parameters for PLA degradation as detected by this kit. Therefore, a time course of PLA degradation was performed to determine when one unit (releases 1 μM of substrate per minute) of Proteinase K saturates the detection limit of the L-lactate detection kit. After 240 min of incubation, saturation of detectable L-lactate in the supernatant was observed (Figure 1B). Thus, incubations for 24 h were adequate to measure saturated degradation of PLA by this purified hydrolase.

Next, assay conditions were altered to mimic expected variables in media conditions during microbial growth. Altering the temperature between 21°C and 37°C had no effect on the ability of Proteinase K to degrade PLA (Supplementary Figure 1). Next, acidification of the media or an addition of carbon was investigated to see if it could alter the sensitivity of our L-lactate detection kit. No significant differences were found between the release of L-lactate in more acidified media, and no differences were seen in lactate levels with the addition of glucose (Supplementary Figures 2, 3). Switching Proteinase K buffer to MSM had no negative effect on enzymatic activity and led to a decrease in background detection of L-lactate. Furthermore, L-lactate release from PLA in the presence of Proteinase K was consistent in biologically relevant levels of salinity (Supplementary Figure 4). The results of these initial assays gave us confidence in our positive control, Proteinase K, for measuring PLA degradation. Finally, laboratory conditions of pH, temperature and salinity that microbes would be exposed to did not significantly alter L-lactate release by Proteinase K.

### Isolation of a Soil Bacterium, *Bacillus pumilus* B12, Capable of Degrading Poly-Lactic Acid Films

**FIGURE 2:**
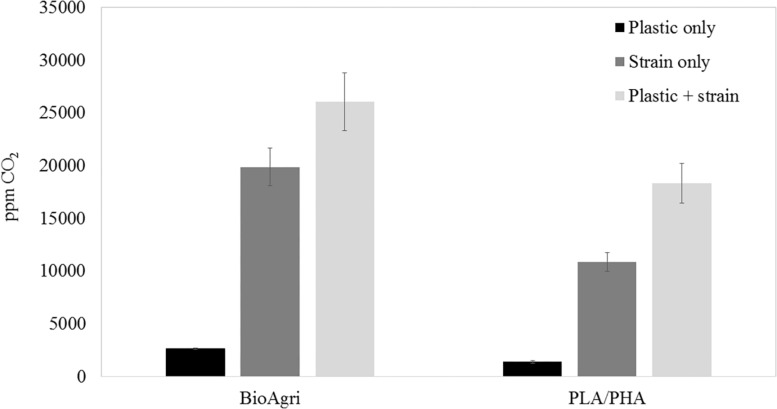
Headspace CO_2_ concentrations after 16 days of culturing *Bacillus pumilus* B12 in MSM with squares of biodegradable plastic mulch films. Data are means and standard deviations of triplicate culture flasks.

**FIGURE 3:**
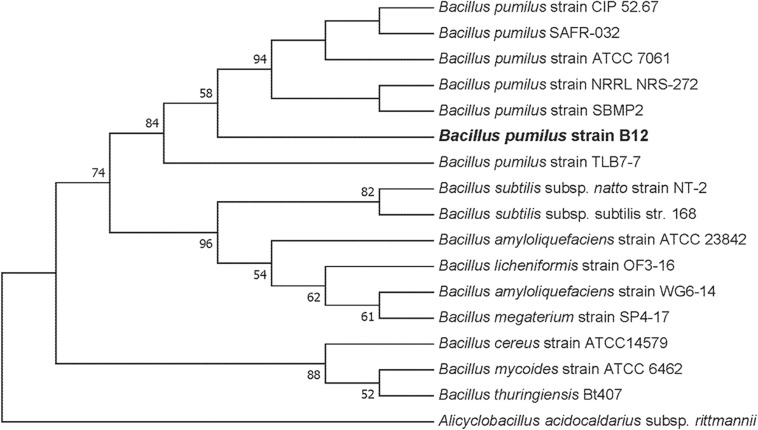
Maximum likelihood phylogenetic tree of 16S rDNA for the environmental isolate B12 compared to other *Bacillus* spp. This bootstrap consensus tree was constructed with 500 replicates. Bootstrap values were used as confidence values for the nodes. Nucleotide sequences for the other bacteria were retrieved from NCBI. The accession numbers are as follows: *Bacillus amyloliquefaciens* (HQ874610.1), *Bacillus megaterium* strain SP4-17 (HQ874615.1), *Bacillus licheniformis* strain OF3-16 (HQ874614.1), *Bacillus amyloliquefaciens* strain ATCC 23842 (EU689157.1), *B. subtilis* strain DSM10 (AJ276351.1), *Bacillus licheniformis* strain HSP-1 (HQ874617.1), *B. subtilis* subsp. natto strain NT-2 (HQ874611.1), *B. subtilis* ATCC 6633 (AB018486.1), *B. pumilus* strain TLB7-7 (HQ874616.1), *B. pumilus* strain ATCC 7061 (AY876289.1), *B. pumilus* strain NBRC 12092 (NR_112637.1), *B. pumilus* strain CIP 52.67 (NR_115334.1), *B. pumilus* strain NRRL NRS-272 (NR_116191.1), *B. pumilus* strain SBMP2 (NR_118381.1), *Bacillus cereus* strain ATCC11778 (AF290546.1), *Bacillus cereus* strain ATCC14579 (AF290547.1), *Bacillus mycoides* strain ATCC 6462 (EF210295.1), *Bacillus thuringiensis* Bt407 (ACMZ01000011.1), and *Alicyclobacillus acidocaldarius* subsp. *rittmannii* (AB089859.1).

To culture PLA degrading microorganisms, samples were collected from agricultural soils where biodegradable plastic mulch films are used and inoculated into enrichment cultures. Soils were diluted and plated on MSM agar containing a square of biodegradable plastic mulch films (BDMs). Three bacterial isolates were collected from the PLA/PHA films and were identified as a *Rhodococcus* sp., *Streptomyces* sp. and *Bacillus* sp. The *Bacillus* sp. isolate (B12) was identified as *B. pumilus* based on 16S rDNA gene sequencing (Figure 3). The *Rhodococcus* sp. sample contained other bacterial species and the *Streptomyces* sp. had weak growth on PLA/PHA films. The *Bacillus* species therefore was carried forward in these experiments. When incubated in MSM with pieces of BDMs, the strain caused a greater increase in CO_2_ in the headspace of the cultures compared to uninoculated controls (Figure 2). Over a 16-day incubation, the mass of plastics was decreased by 9.45% and 2.97% for BioAgri^®^ and PLA/PHA film, respectively. The evolution of CO_2_ combined with mass loss of plastics indicated that this strain had the ability to biodegrade the plastics.

**FIGURE 4:**
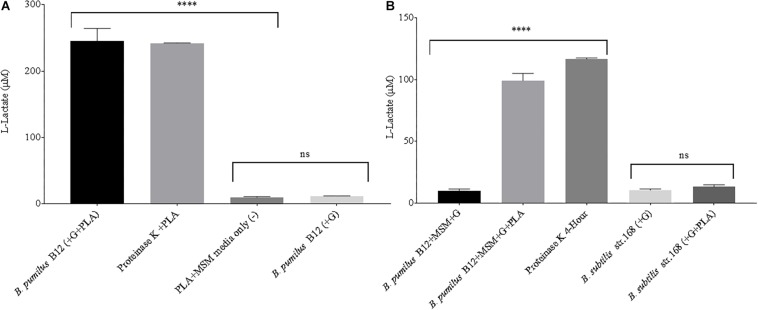
**(A)** High levels of L-lactate can be detected in 3-week microbial cultures when both glucose and PLA are added. *B. pumilus* B12 was incubated for 3 weeks in minimal salt medium with PLA films and a primary carbon source of glucose was either absent (–) or present (+). Results were compared to PLA films incubated with Proteinase K for 4 h, and PLA incubated in MSM as a negative control. ^****^ indicates ANOVA *p*-value ≤ 0.0001 and ns indicates ANOVA *p*-value ≥ 0.05 compared to PLA + MSM media only (–). These assays were performed with three biological and three technical replicates. **(B)** The addition of glucose significantly increases the release of L-lactate from PLA in microbial cultures. All microbial samples were incubated for 48 h in MSM supplemented with glucose (G). Supernatant samples that led to over saturation of the L-lactate detection assay were diluted 1:10 to ensure more accurate results. ^****^ indicates ANOVA *p*-value ≤ 0.0001 compared to *B. pumilus* without PLA and ns indicates *t*-test *p*-value ≥ 0.05 comparing *B. subtilis* str.168 with and without PLA. These assays were performed with three biological and three technical replicates.

Next, supernatant samples of microbial cultures grown in the presence of laboratory-generated PLA films were tested for L-lactate release. All cultures were grown under static conditions, limiting the total biomass created. This allowed us to simulate conditions more commonly found in the soil environment. Following a 3-week incubation, low levels of L-lactate were detected in samples grown with PLA as their sole carbon source. However, when the MSM + PLA cultures were supplemented with glucose, large amounts of L-lactate were detected in the supernatant (Figure 4A). This suggests that this strain can degrade the plastic but generates more esterase activity in the presence of glucose. To confirm that detected L-lactate was not a by-product of glucose fermentation, supernatants from *B. pumilus* cultures grown in glucose media with and without the addition of a PLA film were tested. After just 48 h in minimal L-lactate medium, significantly higher concentrations of L-lactate were detected in cultures supplemented with both glucose and PLA (MSM + G + PLA) than cultures supplemented with glucose alone (MSM + G) (Figure 4B). In fact, the levels of L-lactate detected from MSM + G + PLA cultures were comparable to PLA films exposed to our positive control, Proteinase K, over the same time period. Following this result, all standard media had 55 μM of glucose or another sugar added to prime PLA degradation. No significant release of L-lactate was seen for *B. subtilis* strain 168 (NC_000964.3), suggesting that the active enzymes degrading PLA are either unique to *B. pumilus*, or not expressed in *B. subtilis* in the same conditions (Figure 4B). This evidence also supports the conclusion that the high L-lactate levels detected in the supernatant were a product of PLA degradation and not glucose fermentation by *B. pumilus* B12. This assay is a demonstration that degradation can be detected over a far shorter period (i.e., hours to days) than conventional CO_2_ and film mass loss analysis with glucose priming (i.e., days to weeks).

**FIGURE 5:**
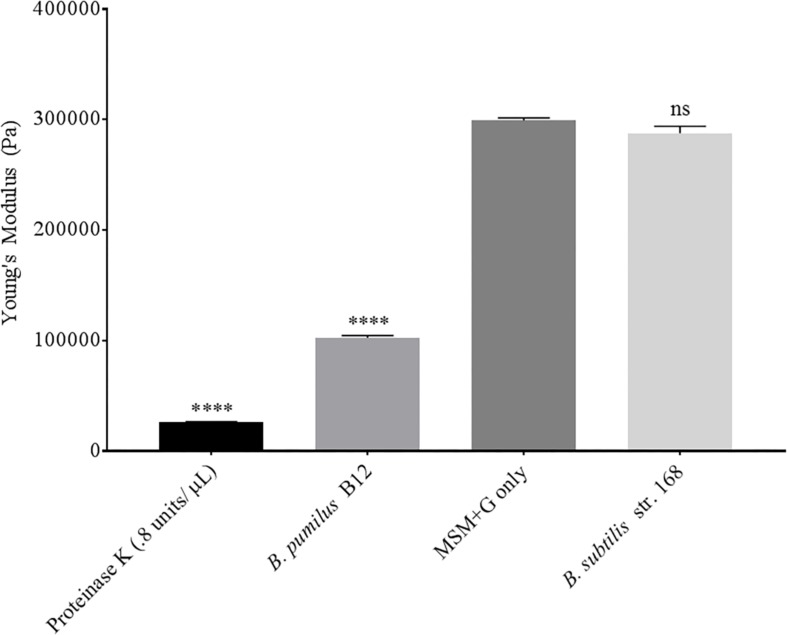
PLA films show a decrease in elastic strength when incubated with *B. pumilus B12.* Shows the average of all cantilever measurements taken over 3 biological replicates. Elastic strength is measured in Young’s modulus Pascals (Pa). All samples were incubated in MSM + glucose for 48 h. *B. subtilis* str.168 (NC_000964.3) was used as a negative control. ^****^ indicates ANOVA *p*-value ≤ 0.0001 and ns indicates ANOVA *p*-value ≥ 0.05 compared to MSM plus glucose (MSM + G) control. These assays were performed with three biological and three technical replicates.

**FIGURE 6:**
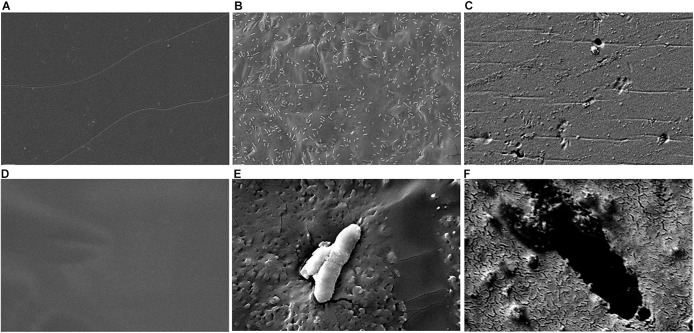
Scanning electron microscopy (SEM) shows PLA fracturing around *B. pumilus* B12 cells. **(A–C)** 5,000× magnification of 10 mg/mL of PLA film for 48 h with: media only **(A)**, *B. pumilus B12* **(B)**, and Proteinase K **(C)**. **(D–F)** 30,000× magnification of 10 mg/mL PLA films with: media only **(D)**, *B. pumilus* B12 **(E)**, and Proteinase K **(F)**.

Although we detected L-lactate release from our PLA films, we wanted an independent confirmation that PLA was being degraded. Thus, we utilized atomic force microscopy (AFM) to detect nano-scale changes in the mechanical properties of the poly-lactic acid films. Following a 48-h incubation, we observed a significant decrease in the Young’s modulus measured in kilopascals (kPa) of PLA films when exposed to our microbial isolate, *B. pumilus* B12, indicating a loss in elastic strength of the polymer (Figure 5). This loss corresponded to an approximately threefold loss in kPA compared to media only control films. To test if the differences seen in PLA elastic strength were caused by activities related to this specific microbe, *Bacillus subtilis* strain 168 (previously shown to not release L-lactate from PLA films, Figure 4B) was used as a negative control. No significant differences in the Young’s modulus of the PLA could be detected when incubated with *B. subtilis* str. 168 in MSM + glucose (Figure 5). This indicates the loss of tensile elasticity of the plastic film is specifically due to the presence of *B. pumilus* B12. Next, SEM was utilized to visualize *B. pumilus* B12 adhesion and growth on PLA plastic films. While observing the bacterial growth on PLA, several fractures were seen in the plastic at both 5,000× and 30,000× magnification (compared to controls, Figures 6A,D) that correlated with the presence of *B. pumilus* B12 cells (Figures 6B,E). These fractures shared similar features to those observed on PLA with Proteinase K addition (Figures 6C,F). However, the proteinase K caused greater damage to PLA than we observed from the bacteria. This is reflected in levels of PLA release and AFM elasticity (Figures 4, 5). The combination of these fractures and the loss of elastic strength of the plastic film are in concordance with our L-lactate detection assays and provide strong evidence that *B. pumilus* B12 is degrading the PLA films and degradation is detectable within 48 h.

### Carbon, Organic Nitrogen, Phosphate, and Potassium Concentrations in Media Regulate Microbial Degradation of Polylactic Acid

**FIGURE 7:**
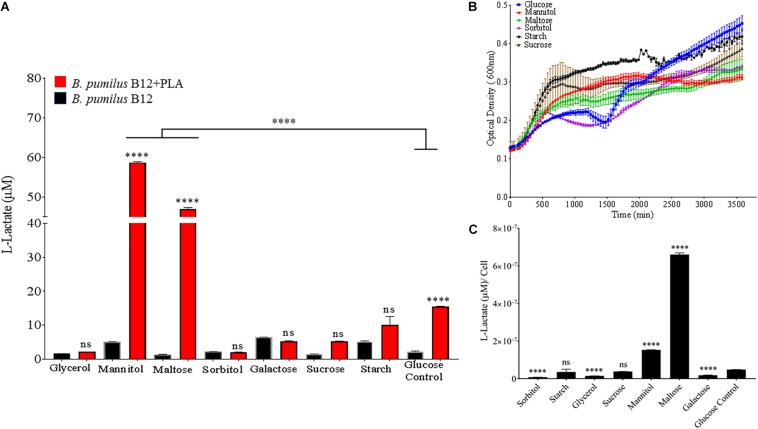
*Bacillus pumilus* B12 increases alters L-lactate release from PLA when grown in different carbon sources. **(A)** Initial measurements of *B. pumilus* B12 grown in different primary carbon sources at a concentration of 55 μM taken after 48 h. Red bars indicate the addition of PLA, black bars indicate B12 grown in media only. All ^****^ and ns above red bars indicates ANOVA ^****^p-value ≤ 0.0001; ns, not significant against media only control. ^****^ above the line indicates *p*-value ≤ 0.0001 against glucose carbon source. These assays were performed with three biological and three technical replicates. **(B)** Optical density-based growth curves of *B. pumilus* B12 in the carbon sources taken over 48 h. Each growth curve represents twelve biological replicates. **(C)** Release of L-lactate from PLA normalized for growth of *B. pumilus* B12 after 48 h. ANOVA results ^****^p-value ≤ 0.0001 ns, not significant against standard glucose supplementation.

In order to identify environmental conditions that regulate degradation of poly-lactic acid by these microbes, we altered carbon, nitrogen, phosphate and potassium sources, as these are some of the most important nutrients found in the soil environment and are known to regulate microbial growth and enzymatic expression (Newman et al., 2016). Since supplementation with glucose increases PLA degradation (Figures 4A,B), we investigated the effects of altering the primary carbon source on the ability of *B. pumilus* B12 to degrade PLA. Following a 48-h treatment with a variety of carbon sources, we saw significant increases in L-lactate release from PLA when changing the primary carbon source from glucose to the disaccharide maltose and the sugar alcohol mannitol (Figure 7A). Reductions in L-lactate release were seen for two non-fermentable carbon sources, glycerol and sorbitol, as well as galactose, however, none of these reductions were significant compared to the same grow conditions lacking PLA (Figure 7A). To determine if these changes were the result of altered growth rates, we determined the amount of L-lactate detection per cell to help visualize the effect of carbon sources on PLA degradation. To accomplish this, we performed 48-h growth curves in each of the different carbon sources to determine the approximate concentration of cells at the time of L-Lactate measurement (Figure 7B). After taking cell numbers into account, both maltose and mannitol were shown to have a significant increase in PLA L-lactate released per cell compared to glucose (Figure 7C). Furthermore, sorbitol, glycerol and galactose all showed significant reductions when taking cell number into account (Figure 7C).

**FIGURE 8:**
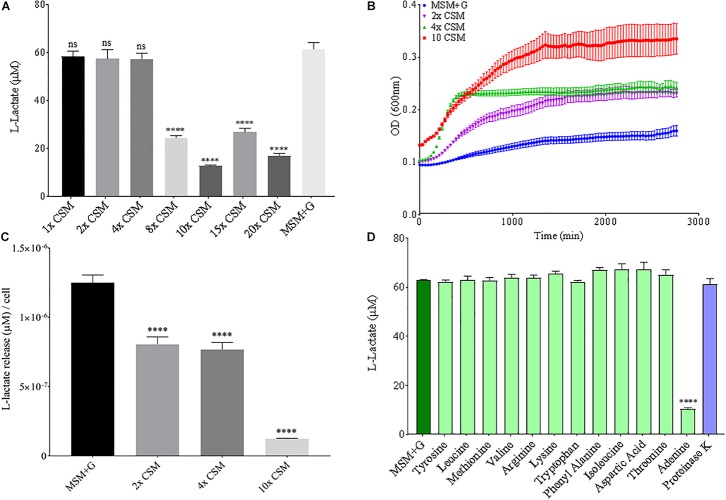
Increasing adenine concentrations available to *B. pumilus* B12 leads to a decrease in L-Lactate release from PLA. **(A)** L-lactate release after 48 h from PLA incubated with *B. pumilus* B12 supplemented with increasing concentrations of complete supplement media (CSM). ^****^ indicates ANOVA *p*-value ≤ 0.0001 and ns indicates ANOVA *p*-value ≥ 0.05 against MSM plus glucose (MSM + G) control. These assays were performed with three biological and three technical replicates. **(B)** 48-h growth curves of *B. pumilus* B12 with added concentrations of CSM. Each growth curve represents twelve biological replicates. **(C)** Release of L-lactate from PLA normalized for growth of *B. pumilus* B12 after 48 h. ^****^ indicates ANOVA *p*-value ≤ 0.0001 against MSM + G. **(D)** L-lactate release after 48 h from PLA incubated with *B. pumilus* B12 with single amino/nucleic acid supplementations. All supplementations are 10-fold higher than the standard concentration found in complete supplement media. Results have been normalized to account for L-lactate released from nutrient supplementation alone. ^****^ indicates *p*-value ≤ 0.0001 against MSM + G as tested by one way ANOVA compared to MSM + G. These assays were performed with three biological and three technical replicates.

Since nitrogen is often limiting in soils, and thus, is added to agricultural soils as fertilizer, both nitrogen source and concentration were altered to see the effect on *B. pumilus* B12 PLA degradation. No notable changes or trends in PLA degradation were observed when increasing the concentration of ammonium sulfate in the media or for sodium nitrate (Supplementary Figures 5, 6). Additionally, no noteworthy changes or trends in L-lactate release were observed when increasing the total nitrogen concentration with nitrate (Supplementary Figure 5). Thus, we tested the effects of changing the nitrogen source through the addition of free amino acids. Complete supplement media (CSM); which contains most amino acids and adenine, was added to the MSM medium and plastic degradation was quantified through L-lactate release. No significant changes in L-lactate were observed with standard additions of CSM, however as concentrations increased, the amount of L-lactate released by *B. pumilus* B12 + CSM + PLA cultures decreased (Figure 8A). Adding these amino acids slightly increased the overall growth of *B. pumilus* B12, indicating that the decrease seen in PLA degradation correlates with the microbe’s physiology and not its overall abundance (Figures 8B,C). To determine if specific amino acids drove this reduction in PLA degradation, individual amino acids or nucleotide bases were added as supplements, and we observed that the reduction in L-lactate release was not being driven by any of the free amino acids in the supplement, but instead the nucleotide base adenine (Figure 8D). These results show that increasing free nitrogen alone does not drive PLA degradation up or down, but the supplementation of adenine specifically reduces degradation.

**FIGURE 9:**
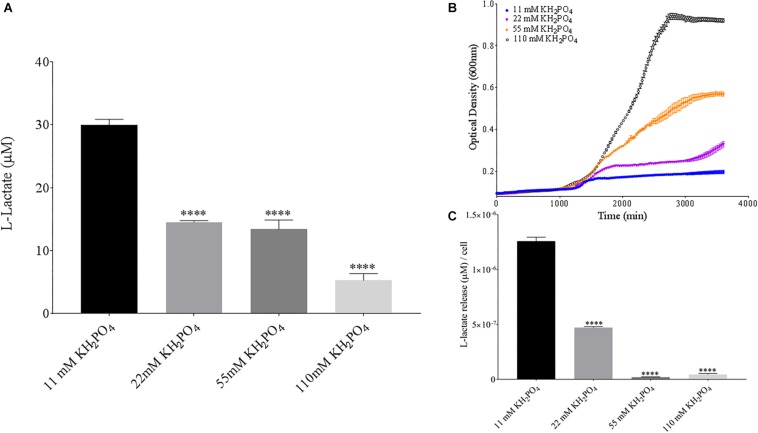
*Bacillus pumilus* B12 decreases L-lactate released from PLA in increased KH_2_PO4. **(A)** Initial measurements of *B. pumilus* B12 L-lactate release from PLA taken after 48 h. KH_2_PO4 concentration increases are based off standard MSM with glucose (MSM + G). These assays were performed with three biological and three technical replicates. **(B)** Optical density-based growth curves of *B. pumilus* B12 in the phosphate sources taken over 50 h. Each growth curve represents eight biological replicates. **(C)** Normalized L-lactate release/cell based on data from **(A,B)**. ^****^ indicates ANOVA *p*-value ≤ 0.0001 compared to MSM + G.

**FIGURE 10:**
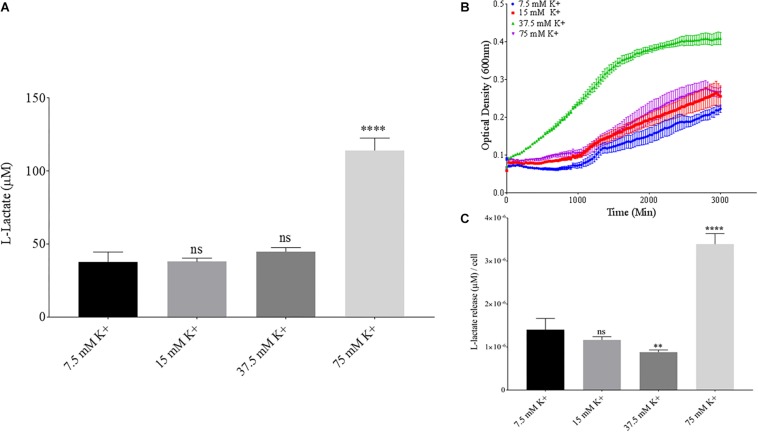
*Bacillus pumilus* B12 changes L-lactate release in K+ when normalized for growth. **(A)** Initial measurements of *B. pumilus* B12 L-lactate release from PLA taken after 48 h. K+ concentration increases are based off standard MSM with glucose (MSM + G). These assays were performed with three biological and three technical replicates. **(B)** Optical density-based growth curves of *B. pumilus* B12 in different K+ concentrations taken over 48 h. Each growth curve represents 8 biological replicates. **(C)** Relative release of L-lactate/cell based on growth after 48 h. ^****^ indicates ANOVA *p*-value ≤ 0.0001, ^∗∗^ indicates ANOVA *p*-value ≤ 0.01, and ns indicates ANOVA *p*-value ≥ 0.05 against MSM + G (7.5 mM K+).

**FIGURE 11:**
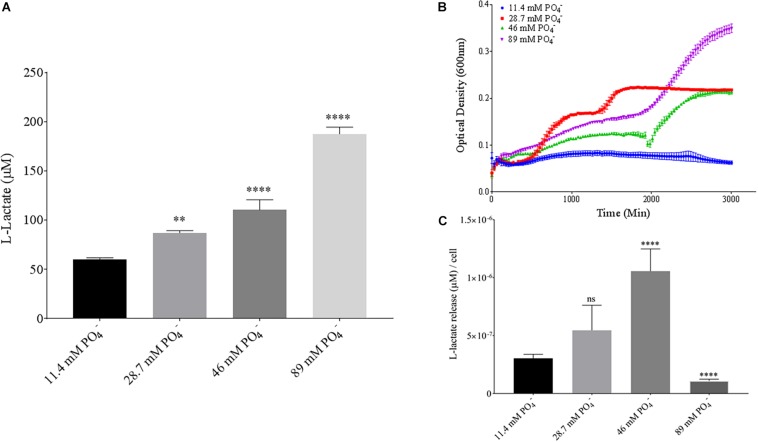
*Bacillus pumilus* B12 changes L-lactate release in PO_4_^–^ when normalized for growth. **(A)** Initial measurements of *B. pumilus* B12 L-lactate release from PLA taken after 48 h. PO_4_^–^ concentration increases are based off standard MSM with glucose (MSM + G). These assays were performed with three biological and three technical replicates. **(B)** Optical density-based growth curves of *B. pumilus* B12 taken over 48 h in different PO_4_^–^ concentration. Each growth curve represents 8 biological replicates. **(C)** Relative release of L-lactate/cell based on growth after 48 h. ^****^ indicates ANOVA *p*-value ≤ 0.0001, ^∗∗^ indicates ANOVA *p*-value ≤ 0.01, and ns is *p*-value > 0.05, all compared to MSM + G control.

Phosphate is an essential nutrient, with free phosphate being limited in the soil environment. Fertilizers work to replenish phosphate depleted soils, having a large impact on the phosphate concentrations available for the soil microbiome (Bargaz et al., 2018). Phosphate and potassium concentrations were changed in the MSM media to see if there would be a change in PLA degradation by *B. pumilus* B12. Potassium is a major cation which has been shown to change kinetics, energy coupling and regulation in microbial life. Both ions have been shown to modulate gene regulation and are possible regulators in the release of enzymes with PLA degradation activity (Botella et al., 2011; Schramke et al., 2017). When increasing potassium phosphate monobasic (KH_2_PO_4_) concentrations in the media, small but significant decreases were initially seen in L-lactate release (Figure 9A). However, there were stark increases in microbial growth with greater concentrations of potassium phosphate (Figure 9B). After normalizing for cell number, we observed a large dose dependent decrease in L-lactate release with higher potassium phosphate levels (Figure 9C). Thus, we see effects by a molecule that contains both potassium and phosphate. To separate the contributions of each ion, media was supplemented with either potassium chloride (KCl) or sodium phosphate (NaPO_4_). We further controlled for sodium and chloride ions by supplementing with sodium chloride, which had no effect on L-lactate release by *B. pumilus* B12 (Supplementary Figure 7). Thus, we were able to measure the contributions of K^+^ and PO_4_^–^ individually. Increasing the concentration of potassium had no initial effect on L-lactate release by *B. pumilus* B12, however as the concentration reached 75 mM, there was a large increase in L-lactate detection (Figure 10A). This increase culminated with a sharp decrease in the growth rate compared to concentrations of 37.5 mM K+ or below. When normalized for cell number, degradation is reduced at 37.5 mM K+, but increases sharply at 75 mM K+ (Figures 10B,C). The significant increase in L-lactate release from PLA correlates with the lower growth rate suggesting added stress on *B. pumilus* B12 at high concentrations of potassium chloride. When altering concentrations of free PO_4_^–^ (without increasing potassium), we see a concentration dependent increase in the total L-lactate being released from PLA films incubated with *B. pumilus* B12 (Figure 11A). However, *B. pumilus* B12 has a higher growth rate in the PO_4_^–^ supplemented media and therefore when the release of L-lactate is normalized for microbial growth, there is a significant decrease in L-lactate release at the highest concentration, when compared to no phosphate controls (Figures 11B,C) although it does increase at lower concentrations. The potassium and phosphate results show a general trend that when *B. pumilus* B12 is growing more efficiently on specific media, less L-lactate is released from PLA films. Conversely, when microbial growth is stressed by being in minimal media or inhibitory concentrations of K+, L-lactate release increases.

## Discussion

The measurement of poly-lactic acid (PLA) degradation has historically relied on techniques that are either time consuming or expensive to perform when running experiments with many variables. This limits the investigatory capabilities into large ranges of changing conditions that PLA is exposed to in different degradation settings. In this study we have shown the ability to measure short term degradation of PLA films from a novel *B. pumilus* isolate. The assay was stable under biologically relevant parameters, with L-lactate concentrations released from PLA mirroring other quantitative and qualitative measurements of degradation (e.g., CO_2_ evolution or film mass loss). Furthermore, the assay may be able to measure L-lactate release from samples grown in anaerobic conditions, although this needs to be more thoroughly tested. The rapid nature of the assay allowed for more robust insight into how changing conditions in microbial growth have large impacts on how *B. pumilus* B12 degrades PLA.

*Bacillus pumilus* B12 showed degradation of PLA primarily when there was a supplementation of carbon in the medium, indicating that *B. pumilus* B12 will degrade PLA even if it is not using it as a sole carbon source. This suggests that hydrolytic activity against PLA is constitutive when scavenging for other nutrient and carbon sources in the environment. This is further supported by *Bacillus pumilus* B12 not growing on PLA films alone, but only growing on PLA/PHA and other polymer blends. This data supports the notion that PLA degradation is occurring via a co-metabolism mechanism; with degradation of the polymer being dependent on the presence of a primary carbon source.

Although glucose supplementation allowed for higher amounts of detectable PLA degradation, the general trend in our study suggests that the more amenable the conditions for growth, the less PLA degradation occurs. The two carbon sources which increased L-lactate detection significantly, mannitol and maltose, both lowered the total biomass produced by *B. pumilus* B12 at the 48-h timepoint. The same trend can be seen for the increases of lactate degradation that occurred in response to specific concentrations of KH_2_PO_4_, K+, and PO_4_^–^ in the media. As growth increased, the relative L-lactate released was reduced. Also, as high concentrations of K+ in the media started to impede growth, there was once again an observable increase in detectable L-lactate released from PLA. This data leads to the hypothesis that in more favorable, free nutrient conditions, *B. pumilus* B12 alters gene expression to reduce extracellular enzymes scavenging for alternative nutrient sources, inhibiting the transcription of hydrolytic enzymes.

When investigating a change in nitrogen sources/concentrations in the media, there was only an impact with adenine supplementation. This combined with the lack of alteration in PLA degradation with increased amino acids, ammonium and nitrate availability suggest that adenine alone is driving a transcriptional change in *B. pumilus* B12. The specificity to adenine over similar or traditionally favored sources of nitrogen downplays the notion of a nutrient driven response. This suggests adenine is acting as specific ligand for a purine riboswitch, negatively regulating downstream enzymes involved in PLA degradation. Riboswitches have been shown to both repress and increase gene expression in *B. subtilis* strains, suggesting similar functions in *B. pumilus* B12 (Christiansen et al., 1997; Mandal and Breaker, 2004).

Overall, this study is the first step into exploring polymer degradation by *B. pumilus* B12 and how degradation changes based on the environment it inhabits. In future work, we will be focusing on both the regulatory mechanisms involved in *B. pumilus* B12 PLA degradation as well as the enzymes(s) acting upon the polymer. Understanding how the bacterium adapts to changing environments will lead to insight into why degradation rates change in real-world settings. Furthermore, this will yield solutions into synthetically altering the physiological machinery of microbial degradation. Identifying active enzymes(s) against PLA will open biochemical avenues of investigation. Both genetic and chemical modifications to active enzymes combined with controlling regulatory mechanisms can lead to a more effectual microbial polymer degrader.

## Data Availability Statement

The datasetsgenerated for this study can be found in the GenBank, accession MN540638, https://www.ncbi.nlm.nih.gov/nuccore/MN540638.

## Author Contributions

KB performed all the enzyme assays, growth assays, and wrote the majority of the manuscript. KB and EP performed the SEM. SH performed AFM. RD, XW, and JD isolated the field isolates and did the original enrichment cultures. EG assisted with 16S phylogeny of the sample. TR directed the project and edited the manuscript.

## Conflict of Interest

The authors declare that the research was conducted in the absence of any commercial or financial relationships that could be construed as a potential conflict of interest.

## References

[B1] Ahn H. K. Huda M. S. Smith M. C. Mulbry W. Schmidt W. F. Reeves J. B. (2011). Biodegradability of injection molded bioplastic pots containing polylactic acid and poultry feather fiber. *Bioresour. Technol.* 102 4930–4933. 10.1016/j.biortech.2011.01.042 21320772

[B2] Arrieta M. P. López J. Rayón E. Jiménez A. (2014). Disintegrability under composting conditions of plasticized PLA–PHB blends. *Polym. Degrad. Stab.* 108 307–318. 10.1016/j.polymdegradstab.2014.01.034

[B3] Bailes G. Lind M. Ely A. Powell M. Moore-Kucera J. Miles C. (2013). Isolation of native soil microorganisms with potential for breaking down biodegradable plastic mulch films used in agriculture. *J. Vis. Exp.* 75:e50373. 10.3791/50373 23712218 PMC3966638

[B4] Bargaz A. Lyamlouli K. Chtouki M. Zeroual Y. Dhiba D. (2018). Soil microbial resources for improving fertilizers efficiency in an integrated plant nutrient management system. *Front. Microbiol.* 9:1606. 10.3389/fmicb.2018.01606 30108553 PMC6079243

[B5] Benson D. Karsch-Mizrachi I. Lipman D. Ostell J. Wheeler D. (2008). GenBank. *Nucleic Acids Res.* 1:33. 10.1093/nar/28.1.15 PMC102453 10592170

[B6] Botella E. Hübner S. Hokamp K. Hansen A. Bisicchia P. Noone D. (2011). Cell envelope gene expression in phosphate-limited *Bacillus subtilis* cells. *Microbiology* 157 2470–2484. 10.1099/mic.0.049205-0 21636651

[B7] Castro-Aguirre E. Iniguez-Franco F. Samsudin H. Fang X. Auras R. (2016). Poly(lactic acid)-mass production, processing, industrial applications, and end of life. *Adv Drug Deliv. Rev.* 107 333–366. 10.1016/j.addr.2016.03.010 27046295

[B8] Christiansen L. C. Schou S. Nygaard P. Saxild H. H. (1997). Xanthine metabolism in *Bacillus subtilis*: characterization of the xpt-pbuX operon and evidence for purine- and nitrogen-controlled expression of genes involved in xanthine salvage and catabolism. *J. Bacteriol.* 179 2540–2550. 10.1128/jb.179.8.2540-2550.1997 9098051 PMC179002

[B9] Edgar R. C. (2004). MUSCLE: multiple sequence alignment with high accuracy and high throughput. *Nucleic Acids Res.* 32 1792–1797. 10.1093/nar/gkh340 15034147 PMC390337

[B10] Emadian S. M. Onay T. T. Demirel B. (2017). Biodegradation of bioplastics in natural environments. *Waste Manag.* 59 526–536. 10.1016/j.wasman.2016.10.006 27742230

[B11] European Bioplastics (2018). “Bioplastics market data 2018: Global production capacities of bioplastics 2018–2023”, in *Proceedings of the 10th European Bioplastics Conference* (Berlin: Nova Institute).

[B12] Fukuzaki H. Yoshida M. Asano M. Kumakura M. Mashimo T. Yuasa H. (1991). In vivo characteristics of high, molecular weight copoly(l-lactide/glycolide) with S-type degradation pattern for application in drug delivery systems. *Biomaterials* 12 433–437. 10.1016/0142-9612(91)90014-2 1909582

[B13] Handtke S. Schroeter R. Jurgen B. Methling K. Schluter R. Albrecht D. (2014). *Bacillus pumilus* reveals a remarkably high resistance to hydrogen peroxide provoked oxidative stress. *PLoS One* 9:e85625. 10.1371/journal.pone.0085625 24465625 PMC3896406

[B14] Hanphakphoom S. Maneewong N. Sukkhum S. Tokuyama S. Kitpreechavanich V. (2014). Characterization of poly(L-lactide)-degrading enzyme produced by thermophilic filamentous bacteria *Laceyella sacchari* LP175. *J. Gen. Appl. Microbiol.* 60 13–22. 10.2323/jgam.60.13 24646757

[B15] Hasim S. Allison D. P. Retterer S. T. Hopke A. Wheeler R. T. Doktycz M. J. (2017). β-(1, 3)-glucan unmasking in some *Candida albicans* mutants correlates with increases in cell wall surface roughness and decreases in cell wall elasticity. *Infect. Immun.* 85:e00601-16. 10.1128/IAI.00601-16 PMC5203643 27849179

[B16] Hayes D. G. Wadsworth L. C. Sintim H. Y. Flury M. English M. Schaeffer S. (2017). Effect of diverse weathering conditions on the physicochemical properties of biodegradable plastic mulches. *Polym. Test* 62 454–467. 10.1016/j.polymertesting.2017.07.027

[B17] Kumar S. Stecher G. Tamura K. (2016). MEGA7: molecular evolutionary genetics analysis version 7.0 for bigger datasets. *Mol. biol. Evol.* 33 1870–1874. 10.1093/molbev/msw054 27004904 PMC8210823

[B18] Li S. Mccarthy S. (1999). Influence of crystallinity and stereochemistry on the enzymatic degradation of poly(lactide)s. *Macromolecules* 32 4454–4456. 10.1021/ma990117b

[B19] Lim H. A. Raku T. Tokiwa Y. (2005). Hydrolysis of polyesters by serine proteases. *Biotechnol. Lett.* 27 459–464. 10.1007/s10529-005-2217-8 15928850

[B20] Link L. Sawyer J. Venkateswaran K. Nicholson W. (2004). Extreme spore UV resistance of *Bacillus pumilus* isolates obtained from an ultraclean Spacecraft Assembly Facility. *Microb. Ecol.* 47 159–163. 10.1007/s00248-003-1029-4 14502417

[B21] Madkour M. H. Heinrich D. Alghamdi M. A. Shabbaj I. I. Steinbüchel A. (2013). PHA recovery from biomass. *Biomacromolecules* 14 2963–2972. 10.1021/bm4010244 23875914

[B22] Mandal M. Breaker R. R. (2004). Adenine riboswitches and gene activation by disruption of a transcription terminator. *Nat. Struct. Mol. Biol.* 11 29–35. 10.1038/nsmb710 14718920

[B23] Masaki K. Kamini N. R. Ikeda H. Iefuji H. (2005). Cutinase-like enzyme from the yeast *Cryptococcus* sp. strain S-2 hydrolyzes polylactic acid and other biodegradable plastics. *Appl. Environ. Microbiol.* 71 7548–7550. 10.1128/aem.71.11.7548-7550.2005 16269800 PMC1287645

[B24] Mihai M. Legros N. Alemdar A. (2014). Formulation-properties versatility of wood fiber biocomposites based on polylactide and polylactide/thermoplastic starch blends. *Poly. Eng. Sci.* 54 1325–1340. 10.1002/pen.23681

[B25] Newman M. M. Lorenz N. Hoilett N. Lee N. R. Dick R. P. Liles M. R. (2016). Changes in rhizosphere bacterial gene expression following glyphosate treatment. *Sci. Total Environ.* 553 32–41. 10.1016/j.scitotenv.2016.02.078 26901800

[B26] Oda Y. Yonetsu A. Urakami T. Tonomura K. (2000). Degradation of polylactide by commercial proteases. *J. Poly. Environ.* 8 29–32. 15468296

[B27] Schallmey M. Singh A. Ward O. P. (2004). Developments in the use of *Bacillus* species for industrial production. *Can. J. Microbiol.* 50 1–17. 10.1139/w03-076 15052317

[B28] Schramke H. Laermann V. Tegetmeyer H. E. Brachmann A. Jung K. Altendorf K. (2017). Revisiting regulation of potassium homeostasis in *Escherichia coli*: the connection to phosphate limitation. *MicrobiologyOpen* 6:e00438. 10.1002/mbo3.438 28097817 PMC5458449

[B29] Singhvi M. Gokhale D. (2013). Biomass to biodegradable polymer (PLA). *RSC Adv.* 3 13558–13568.

[B30] Tomita K. Kuroki Y. Nagai K. (1999). Isolation of thermophiles degrading poly(L-lactic acid). *J. Biosci. Bioeng.* 87 752–755. 10.1016/s1389-1723(99)80148-0 16232549

[B31] Van Dijl J. Hecker M. (2013). *Bacillus subtilis*: from soil bacterium to super-secreting cell factory. *Microb. Cell Fact.* 12:3. 10.1186/1475-2859-12-3 23311580 PMC3564730

