# Supplementary material for: Bacillus pumilus B12 Degrades Polylactic Acid and Degradation Is Affected by Changing Nutrient Conditions
Source: Front Microbiol. 2019 Nov 22;10:2548. doi: 10.3389/fmicb.2019.02548 (PMC6882738; doi:10.3389/fmicb.2019.02548)
Supplement: Supplementary file 1 [file Data_Sheet_1.pdf]

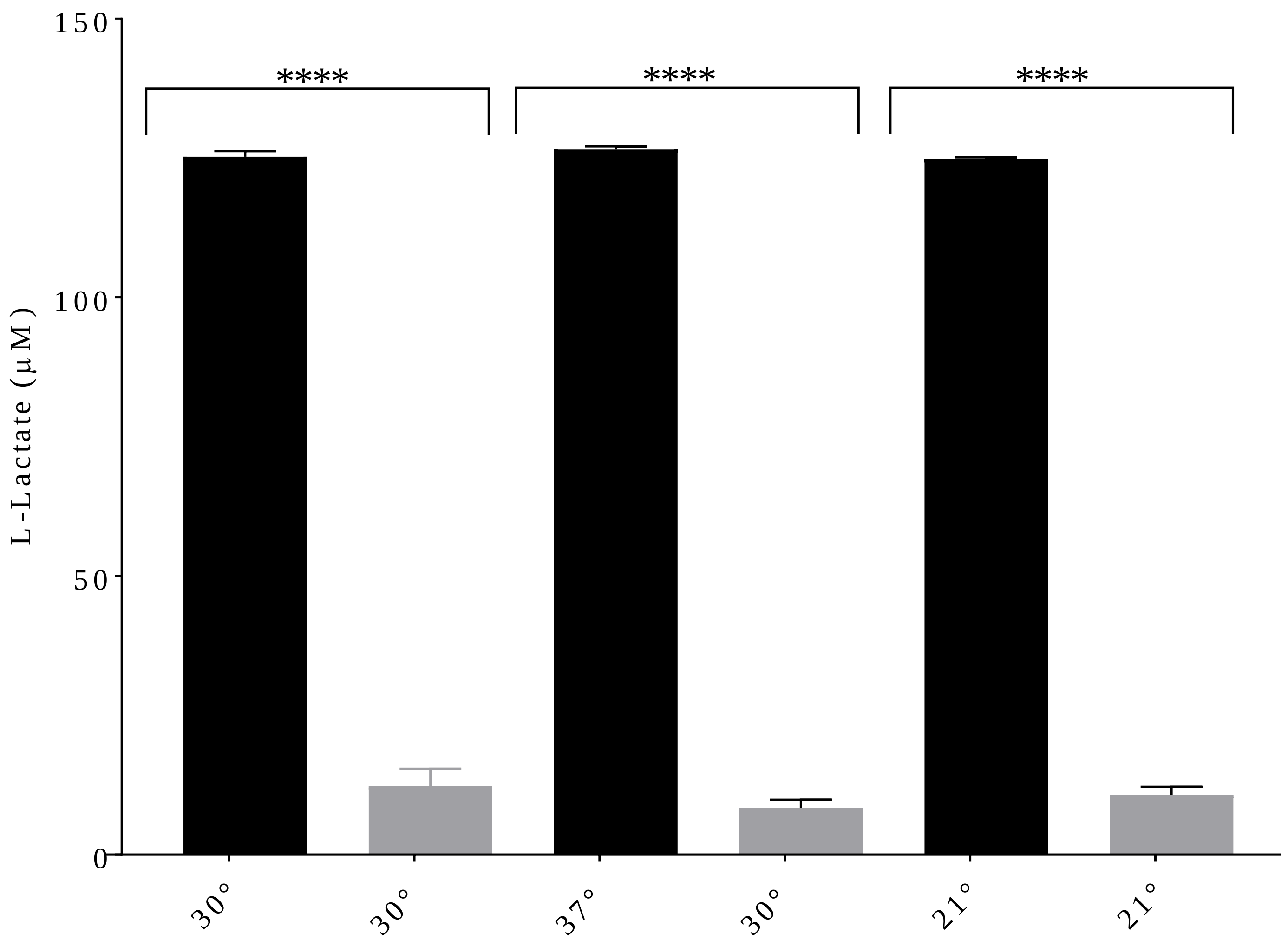

Supplementary Figure 1: Altering the temperature does not have any impact on Proteinase K driven L-lactate release from PLA. 10 mg/mL PLA films of PLA were incubated with (Black) or without (Grey) 1 unit of Proteinase K for 24 hours. ******** indicates t-test p-value ≤ .0001 against Proteinase K treatment at same temperature.

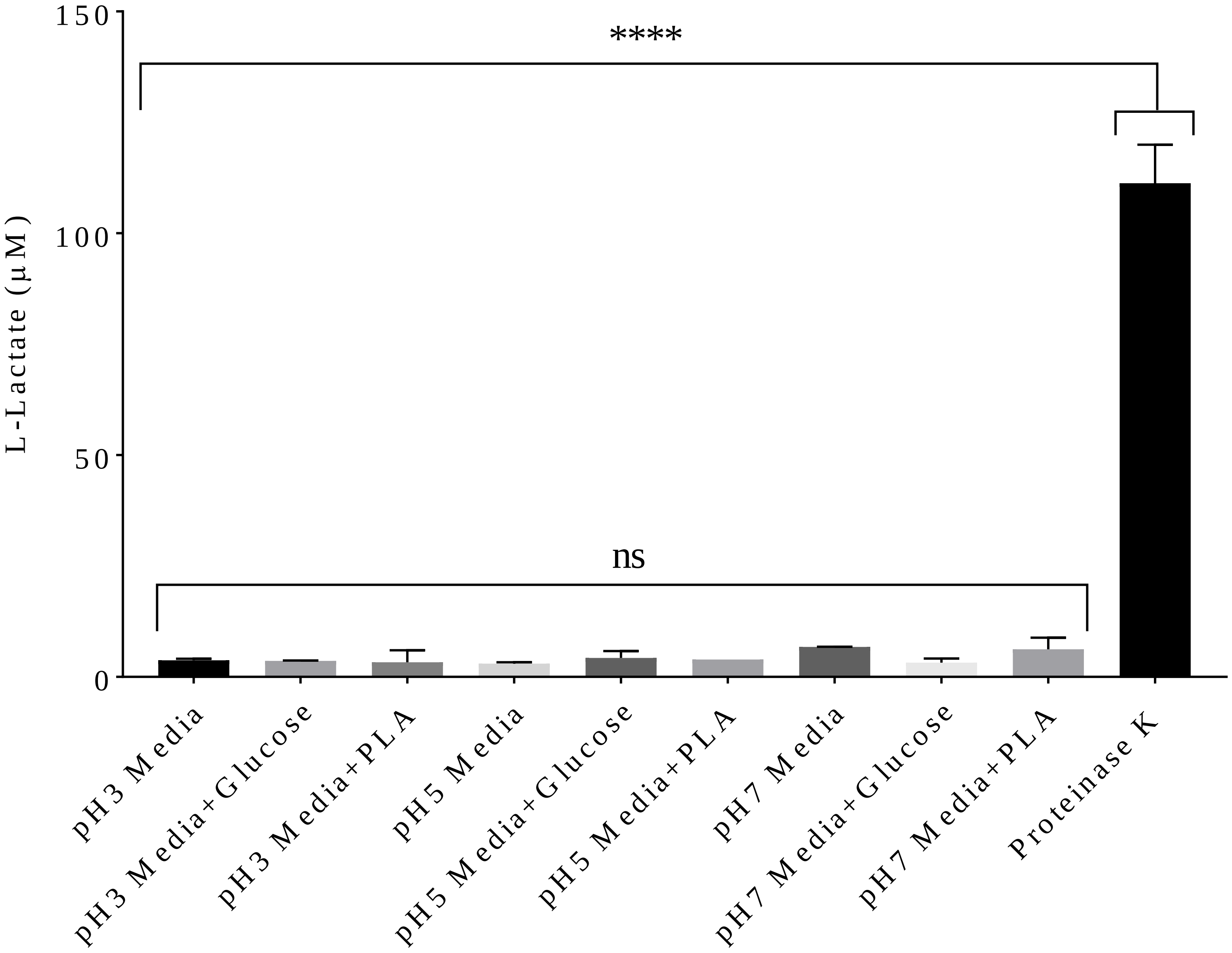

Supplementary Figure 2: Changing the pH of the media does not change detection of L-lactate from PLA films. 10 mg/mL films were incubated for 48 hours in minimal salt media and L-lactate was measured from the supernatant. The pH was adjusted by adding hydrochloric acid to the media prior to incubation. Results are compared to PLA films incubated with our positive control Proteinase K for 24 hours. **** indicates ANOVA **p-value** ≤.0001 and **ns** indicates ANOVA p-value ≥ .05.

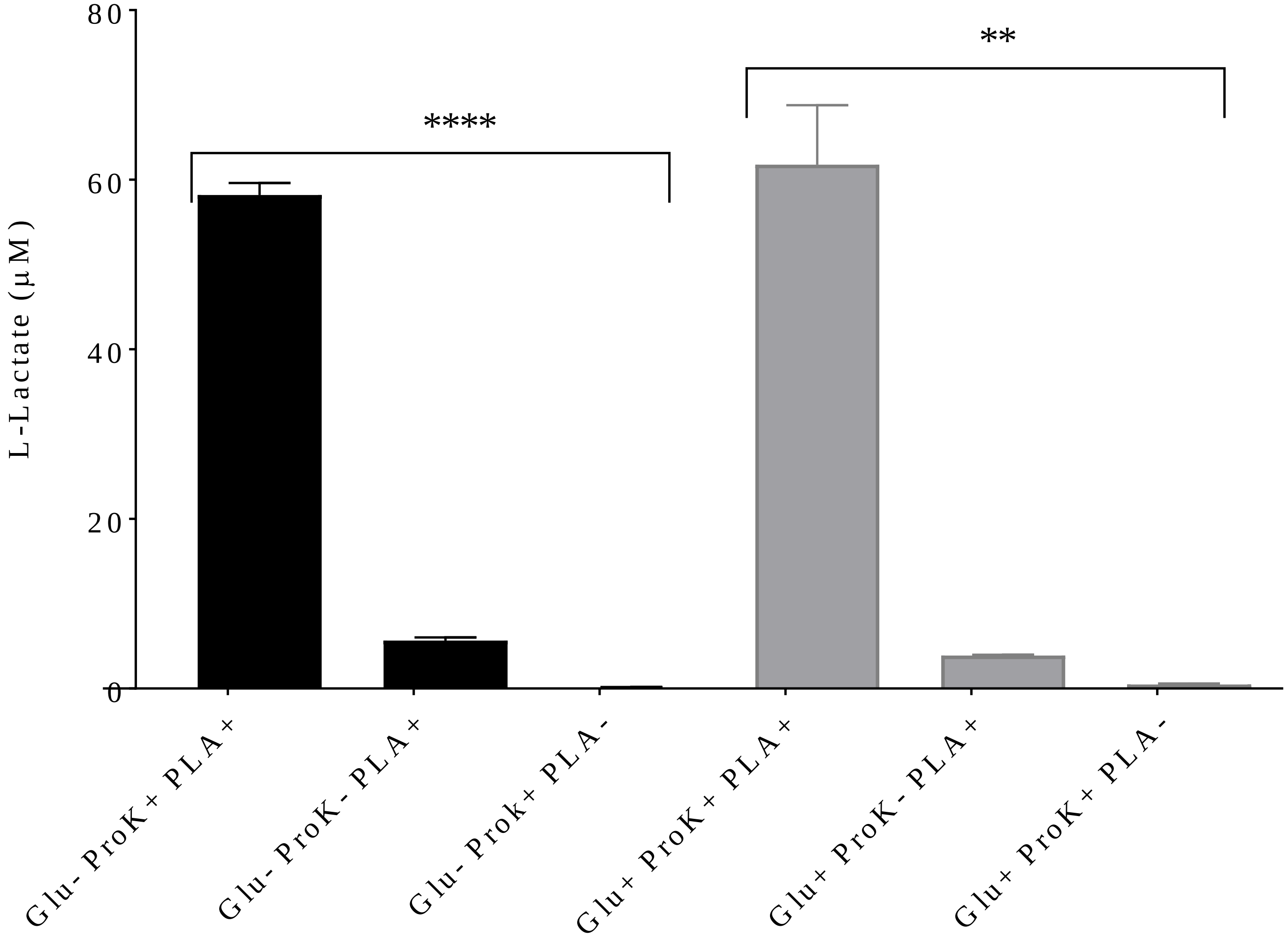

Supplementary Figure 3: Adding glucose has no effect on the efficacy of the L-lactate assay. Black bars represent incubation of minimal salt media (Glu-) and grey bars indicate supplementation with 55 mM glucose (Glu+). Presence or absence of Proteinase K (ProK) and Polylactic acid (PLA) are represented by + and – respectively. ** and **** indicates ANOVA p-value of $\leq .01$ and $\leq .0001$ respectively.

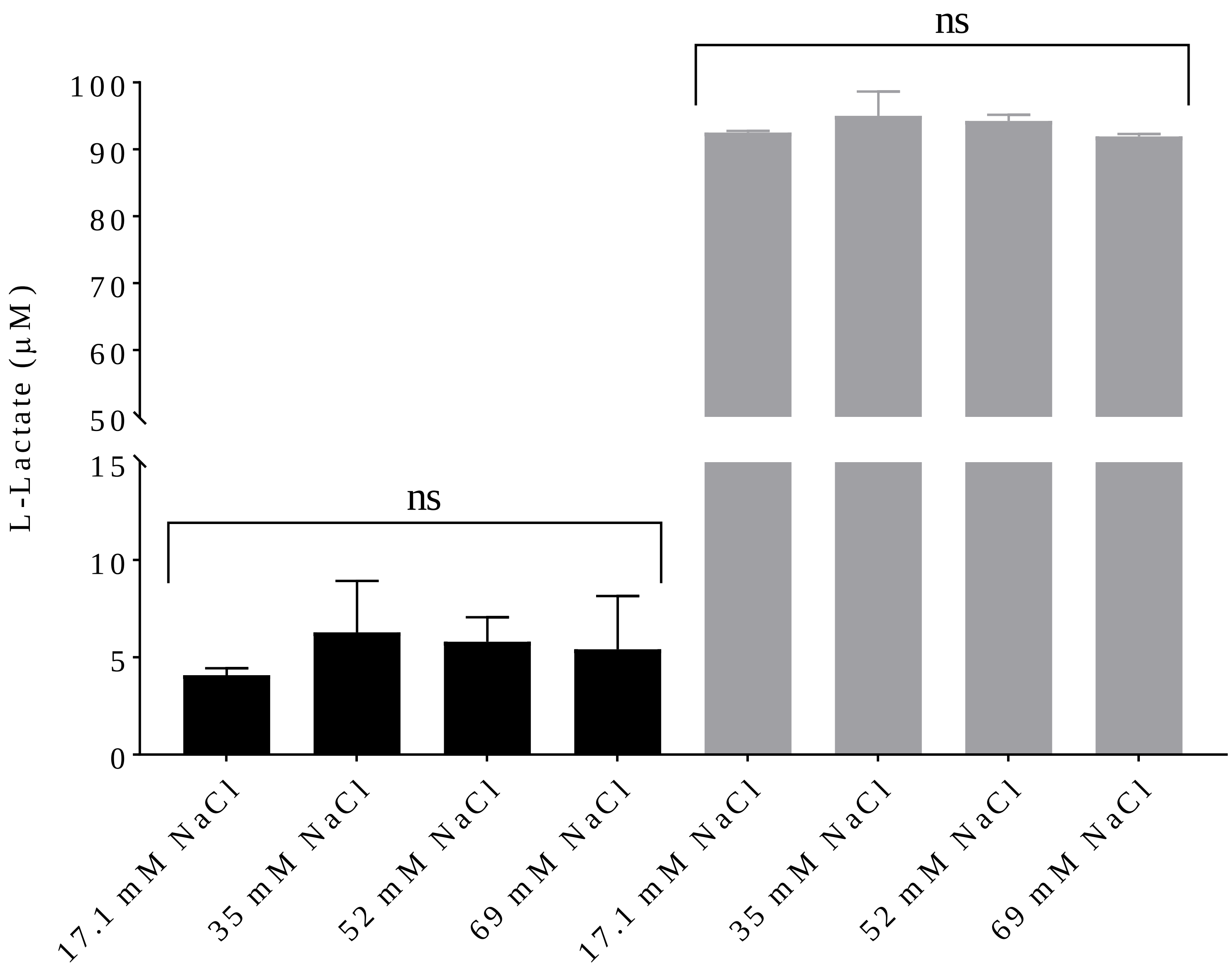

Supplementary Figure 4. Increasing salinity has no effect on L-lactate released from Proteinase K. 10 mg/mL PLA films were incubated for 24 hours in Minimal salt media with glucose (MSM+G) and L-lactate release was measured from the supernatant. Black bars indicate media alone and grey bars indicate 1 unit of Proteinase K addition. "**ns**" indicates ANOVA p-value ≥ .05 against MSM+G control (17.1 mM NaCl).

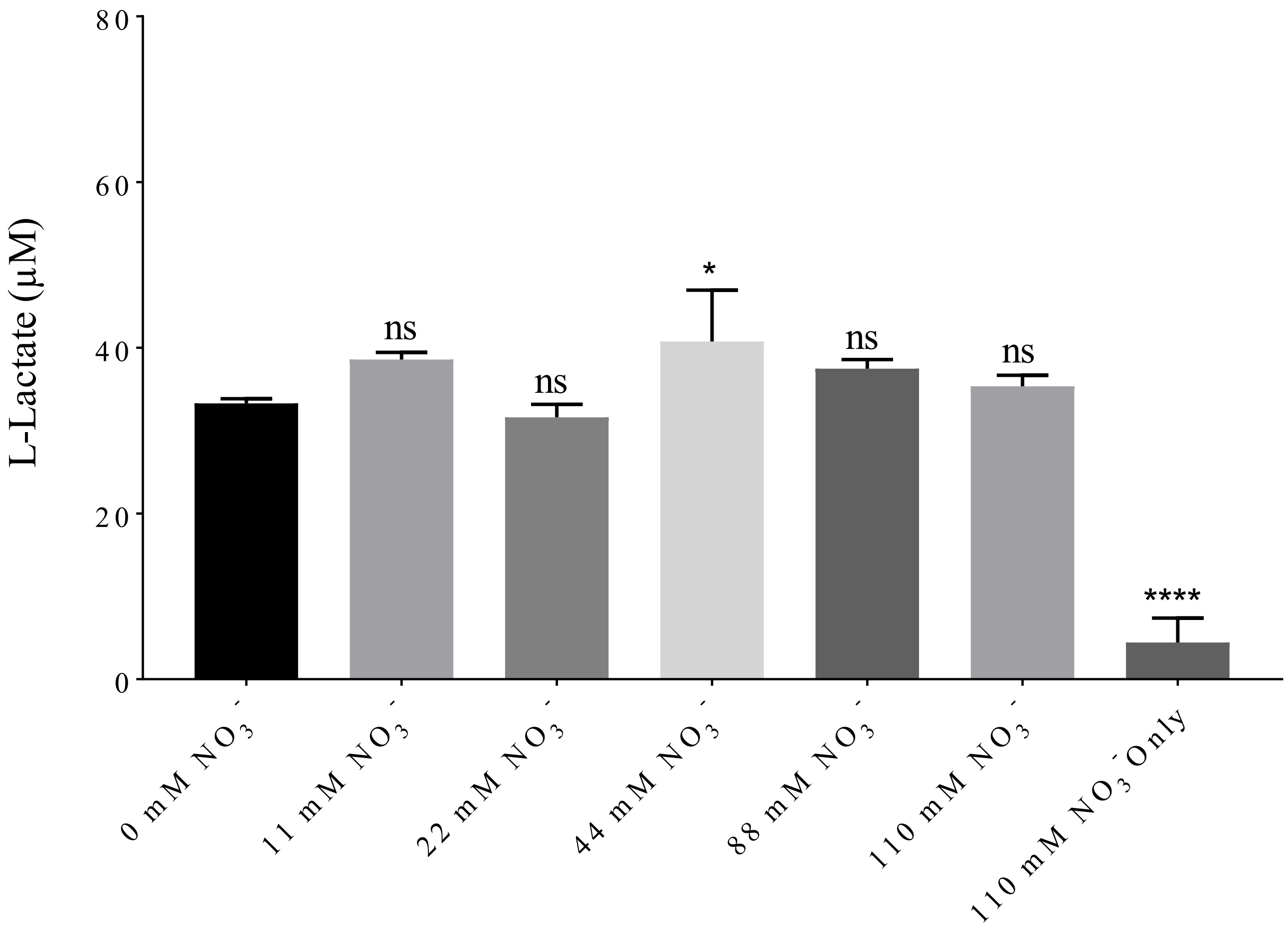

Supplementary Figure 5: Changing primary nitrogen source to Nitrate (NO_3^-) has no effect on PLA degradation by *B. pumilus* B12. PLA films were incubated for 48 hours with *B. pumilus* and supernatant L-lactate measurements were taken. ******** indicates ANOVA p-value $\leq .0001$, ***** indicates ANOVA p-value $\leq .01$ and **ns** indicates ANOVA p-value $\geq .05$ compared to MSM+G control. "NO_3^- only" represents media without bacterial inoculum.

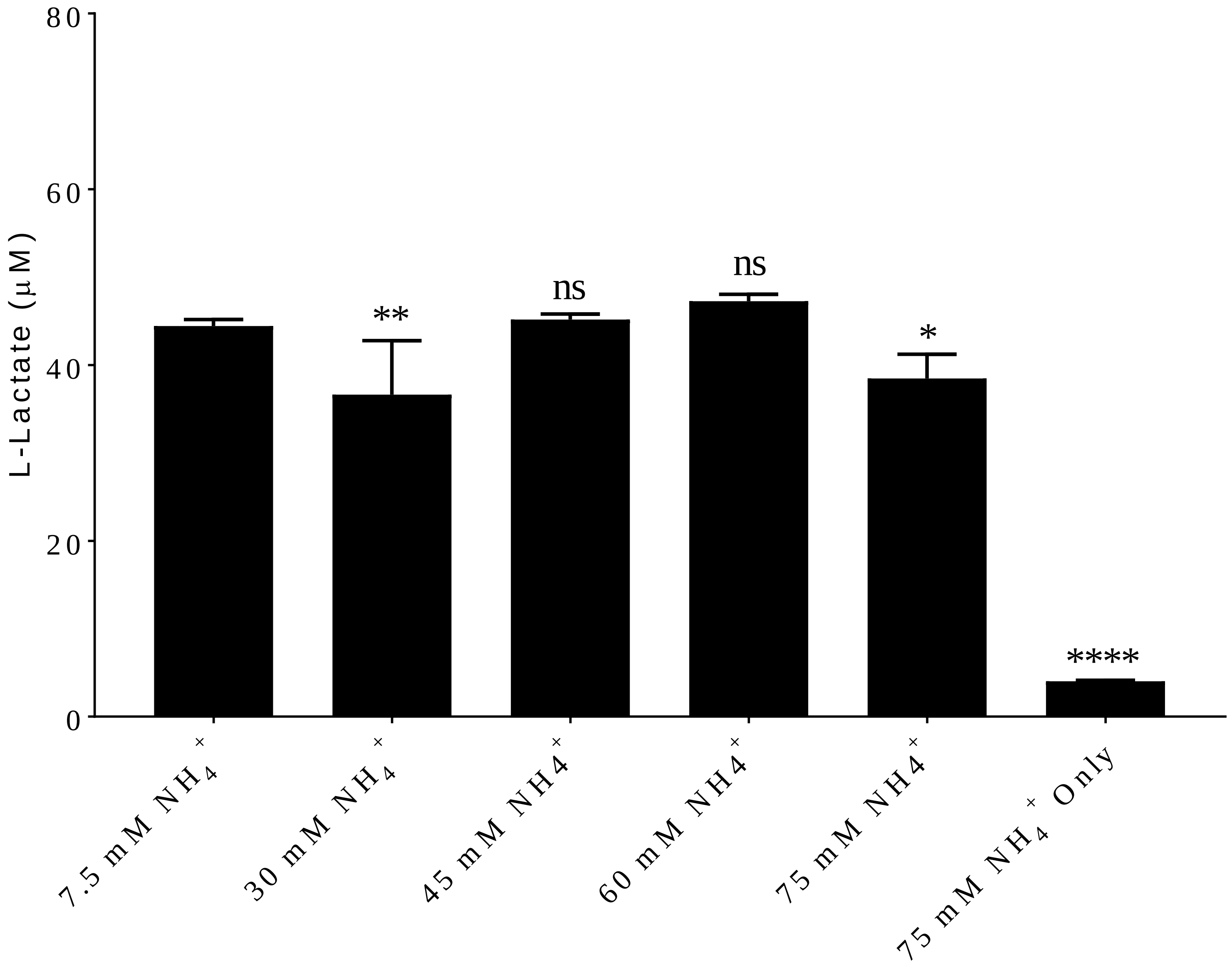

Supplementary Figure 6: Increasing ammonium concentration in the media has no effect on PLA degradation by *B. pumilus* B12. PLA films were incubated for 48 hours with *B. pumilus* and supernatant L-lactate measurements were taken. ******** indicates ANOVA p-value ≤ .0001, ****** indicates ANOVA p-value ≤ .01 , ***** indicates ANOVA p-value ≤ .05 and **ns** indicates ANOVA p-value ≥ .05 against MSM+G control (7.5 mM NH_4^+).

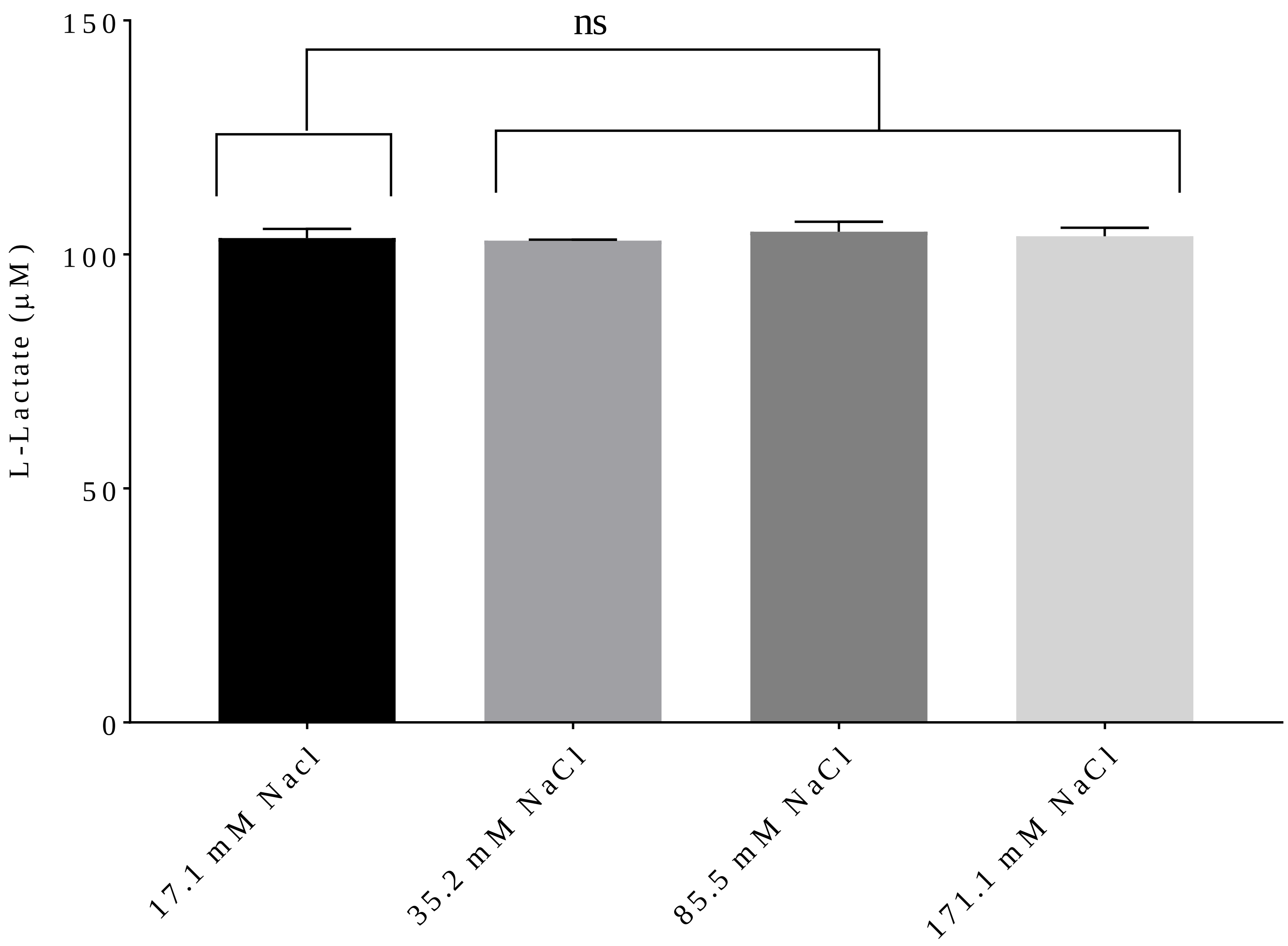

Supplementary Figure 7. increasing NaCl in the media has no effect on PLA degradation by *B. pumilus* B12. Concentrations were increased from the standard NaCl concentration found in minimal salt media (17.1 mM NaCl). **ns** indicates ANOVA p-value ≥ .05 against MSM+G control.
